# New insights into microRNA in dermatological diseases

**DOI:** 10.3389/fmed.2025.1624085

**Published:** 2025-08-11

**Authors:** Mengting Xu, Jiadong Yan, Xin Wang, Yue Zhang, Shengju Yang

**Affiliations:** ^1^Department of Pharmacy, The First People’s Hospital of Zhangjiagang City, Suzhou, China; ^2^Department of Dermatology, Affiliated Hospital of Nantong University, Nantong, China

**Keywords:** microRNA, psoriasis, skin wound, burn, systemic sclerosis, melanoma

## Abstract

MicroRNAs (miRs) are a class of non-coding RNA molecules that regulate gene expression post-transcriptionally. MiRs, as translational repression and/or degradation of target messenger RNAs, are critical regulators of various physiological processes, including cell proliferation, differentiation, death, and immune responses. Currently, miRs are being investigated as potential biomarkers and therapeutic targets for a range of diseases. In recent years, miRs have been reported to be implicated in several pathophysiological processes of dermatological diseases including psoriasis, skin wound, diabetic skin wound, burn, systemic sclerosis, skin tumors (melanoma, squamous cell carcinoma and basal cell carcinoma), recessive dystrophic epidermolysis bullosa, and systemic lupus erythematosus. Mechanistically, the regulation of oxidative stress, inflammation, apoptosis, and angiogenesis may account for the distinct roles of miRs in the skin. A deeper understanding of different miRs and their related regulatory targets is essential for elucidating the pathophysiology of numerous skin diseases. This review briefly summarizes roles and potential applications of miRs within the skin. The combination of miRs with novel materials or compounds may offer innovative approaches for the treatment of skin diseases. However, further research is necessary to facilitate the translation into clinical applications for dermatological diseases.

## 1 Introduction

MicroRNAs (miRs) are key post-transcriptional regulators of gene expression, with emerging roles in dermatological pathophysiology (1). Their tissue-specific expression patterns are particularly relevant in the skin, where miR dysregulation contributes to various dermatological diseases (2–4).

The skin’s accessibility and unique cellular composition, including keratinocytes and fibroblasts, made it an ideal model for studying miR-based therapeutics (5–7). The topical and intradermal delivery of miR modulators, such as nanoparticle-encapsulated antimiRs, is feasible due to the skin’s permeability, which enables targeted therapy for lesions (8, 9). This review focuses on novel insights into miR dysregulation across major dermatological conditions, emphasizing mechanisms linked to oxidative stress, inflammation, apoptosis, and angiogenesis.

This review synthesizes recent advances in miR dysregulation across several dermatological conditions: psoriasis, skin wound, diabetic skin wound, burn, systemic sclerosis (SSc), skin tumors, recessive dystrophic epidermolysis bullosa (RDEB), and systemic lupus erythematosus (SLE). We highlight the following: disease-specific miR signatures and mechanisms, cross-disease roles of key miRs, and the clinical translation of miR-based diagnostics and therapeutics.

Although recent reviews have extensively covered miRs in specific skin diseases, such as the pathogenesis of melanoma (10) and psoriasis (11), this review integrated the cross-disease mechanisms of miRs in oxidative stress, inflammation, apoptosis and angiogenesis, and emphasizes emerging therapeutic strategies, such as miR-nanocornists. They still need further research to translate these findings into clinical practice. It was worth noting that although miRs played a significant role in many other skin diseases, such as photoaging (12), alopecia (13), pigmentation disorders (chloasma, vitiligo, albinism) (14), acne (15), dermatitis (16) and urticaria (17), this review focused on integrating the latest and substantial progress in the above-mentioned specific disease areas to provide a targeted and in-depth analysis.

## 2 Implications of miRs in dermatological diseases

### 2.1 Psoriasis

Psoriasis is a chronic inflammatory skin disease characterized by abnormal differentiation and excessive proliferation of keratinocytes. Psoriasis primarily occurs in adults to severely affect patients’ life quality (18, 19), and is often regarded as an immune-mediated disease model. Factors such as genetic susceptibility, cell cycle regulation, immune response, inflammation, and neurotransmitters may contribute to the development of psoriasis (20, 21). However, the exact mechanisms underlying psoriasis remain to be further elucidated.

Currently, the regulation of miRs in the treatment of psoriasis has garnered significant attention from scientists exploring this field. Research has identified a variety of miRs that influence the development of psoriatic lesions (22, 23). Specifically, 24 miRs were found to be either upregulated or downregulated in keratinocytes from patients with psoriasis, highlighting the importance of miRs in the etiology of psoriasis (24–26). However, the aforementioned study assessed miR levels solely in blood samples rather than in skin lesions. Therefore, further research is necessary to determine whether miRs can serve as biomarkers for psoriasis prognosis or as therapeutic targets for treatment. Additionally, the specificity of miRs to different type of psoriasis also remains unclear.

Dual-tyrosine specificity phosphorylation-regulated kinase 1A (DYRK1A) is a key stabilizer of the epidermal growth factor receptor (EGFR) and plays a crucial role in cytokeratinization, hyperproliferation, aberrant differentiation, and inflammatory infiltration during the development of psoriasis. MiR-215-5p is expressed at low level in psoriasis. Overexpression of miR-215-5p inhibited the proliferation of human keratinocytes (HaCaTs) cell and diminished psoriasis-like inflammation through DYRK1A-mediated regulation of the EGFR signaling pathway (27). In contrast to miR-215-5p, miR-21 is highly expressed in psoriasis (28–30). In HaCaTs, normal human epidermal keratinocytes (NHEKs), and psoriatic skin samples, maternally expressed gene 3 (MEG3) was all dramatically downregulated. Interestingly, MEG3 suppressed proliferation and accelerated apoptosis in activated HaCaT and NHEK cells via miR-21. In detail, miR-21 and MEG3 regulated the expression of caspase-8, cleaved caspase-8, and apoptotic protease activating factor-1 (apaf-1) proteins downstream. These findings demonstrate that the MEG3/miR-21 axis regulates caspase-8 expression, which may contribute to the proliferation and apoptosis of psoriatic keratinocytes (31). In addition, a team investigated the association between the miR-21 binding site single nucleotide polymorphism (SNP) and psoriasis susceptibility in women. They found that SNP rs 4597342 in the 3’ non-coding region of the Integrin Subunit Alpha M (ITGAM) gene affected miR-21 binding, potentially serving as a risk factor for the development of psoriasis. Upregulation of miR-21 expression decreased the production of cluster of differentiation (CD) 11b to disturb macrophage-1 antigen (Mac-1) function, leading to abnormality in innate immune cells, and excess cytokine secretion in the pathogenesis of psoriasis (32). Additionally, miR-21 also mediated angiogenesis, immune response, and apoptosis in psoriasis (33). These findings suggest that miR-21 is a key factor in the etiology of psoriasis and may represent an effective target for treatment.

Similar to miR-21, the up-regulation of miR-744-3p in psoriasis regulates the proliferation and differentiation of keratinocytes by targeting killin (KLLN) (34). Another study found that miR-146b and miR-10b bind directly to the 3’non-coding region (3’-UTR) of the atypical chemokine receptor 2 (ACKR2), suppressing ACKR2 transcription and protein expression in keratinocytes and lymphoid endothelium cells, respectively. When cells are injured, their expression of ACKR2 is further reduced, which serves as a key trigger for the formation of new plaques in many psoriasis patients, a phenomenon known as the Koebner phenomenon (35). These miRs could be valuable targets for therapeutic development in the fight against psoriasis. To identify potential treatment targets, further in-depth studies are needed to enhance our understanding of the role of miRs in the pathogenesis of psoriasis.

Acitretin is the first-line drug for the treatment of psoriasis vulgaris in clinical practice. Previous studies have shown that acitretin can inhibit the mitogen-activated protein kinase (MAPK), Janus kinase (JAK), signal transducer of activator of transcription (STAT) and nuclear factor kappa B (NF-κB) signaling pathways by reducing the expression of specific miRs, thereby alleviating inflammatory responses and keratinocyte proliferation. Acitretin has been found to attenuate the development of psoriasis vulgaris by reducing the levels of miR-146a-5p, miR-21-5p and miR-122-5p (36). MiR-31 is a highly conserved miR, however, there have been few *in vivo* mechanistic studies examining its activity in psoriasis. A research team developed a miR-31 overexpression mouse that exhibited with psoriasis-like skin lesions. It was found that miR-31overexpression significantly led to the upregulation of STAT3 and enhancement of p53, resulting in keratinocytes hyperproliferation. The discovery of the miR-31/STAT3/p53 pathway may provide new approaches for the treatment of psoriasis (37). Additionally, recent studies have demonstrated that miR-17-3p promotes keratinocyte proliferation and pro-inflammatory cytokine secretion by targeting Cln 3-requiring 9 (CTR9), suggesting that miR-17-3p could serve as a novel therapeutic target for psoriasis (38). However, due to the challenges associated with sampling the lesion-free skin from patients with psoriasis, further validation of these findings is necessary.

**FIGURE 1:**
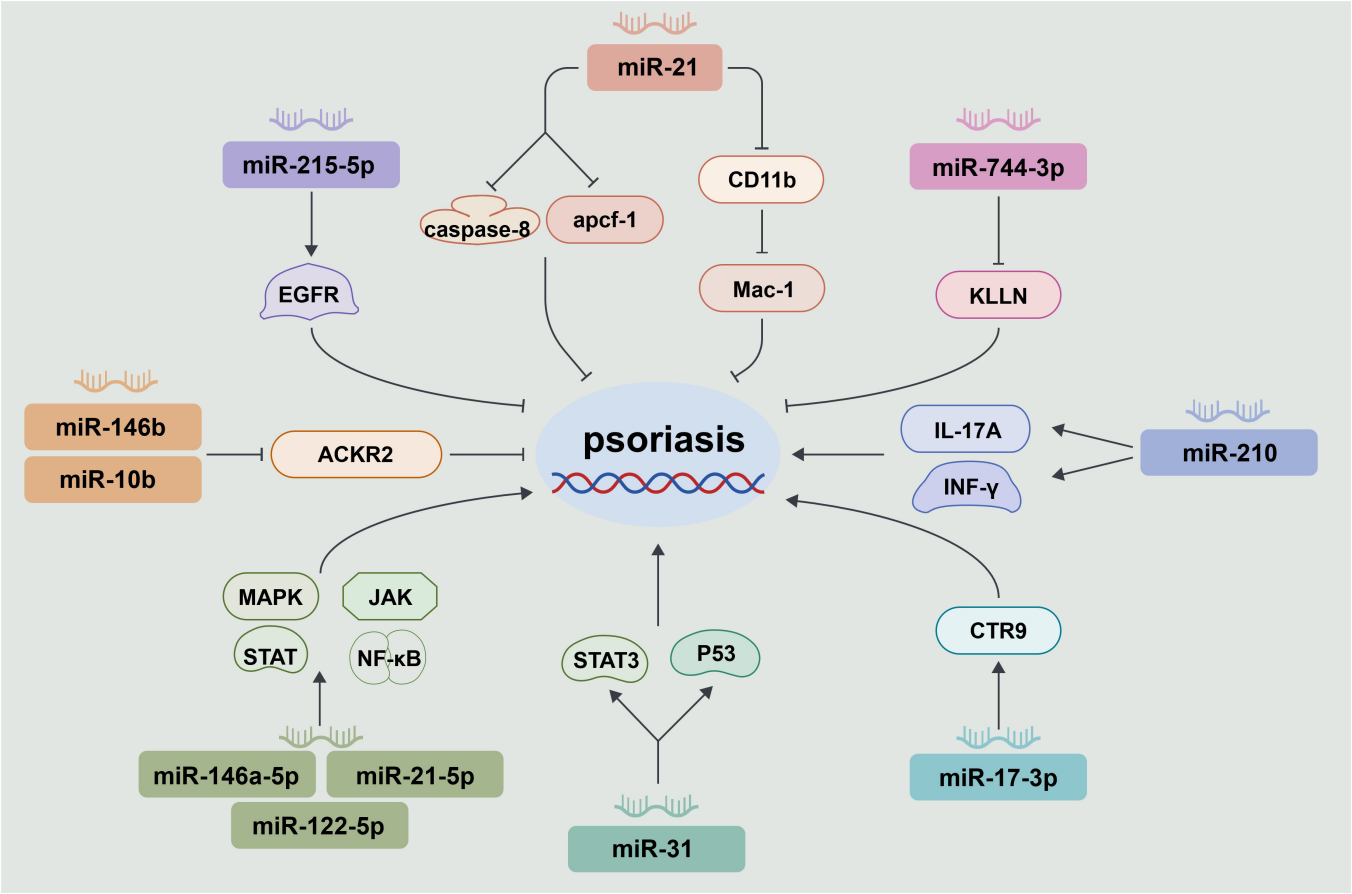
Possible involvement of microRNAs (miRs) in psoriasis. A variety of miRs are involved in regulating the development of psoriasis. Overexpression of miR-215-5p can reduce the proliferation of human keratinocytes (HaCaTs) and alleviate inflammation by inhibiting the epidermal growth factor receptor (EGFR) signaling pathway. MiR-21 promoted the proliferation of keratinocytes and inhibited apoptosis by regulating caspase-8 and apoptotic protease activating factor-1 (apaf-1). It can also interfere with the function of macrophages by affecting the expression of cluster of differentiation (CD) 11b, leading to congenital immune abnormalities. MiR-744-3p targeted killin (KLLN) to promote the proliferation of keratinocytes and inhibit differentiation. MiR-146b and miR-10b, respectively inhibited the transcription and protein expression of atypical chemokine receptor 2 (ACKR2) in keratinocytes and lymphoendothelial cells, thereby reducing the formation of new plaques in patients with psoriasis. Reducing the expression of miR-146a-5p, miR-21-5p and miR-122-5p can inhibit the mitogen-activated protein kinase (MAPK), Janus kinase (JAK), activated signal transducer (STAT) and nuclear factor κB (NF-κB) signaling pathways, thereby alleviating the inflammatory response and keratinocyte proliferation. MiR-31 drived excessive proliferation of keratinocytes by activating STAT3 and enhancing p53 signaling. MiR-17-3p inhibited Cln 3-requiring 9 (CTR9) to promote the proliferation of keratinocytes and the secretion of pro-inflammatory cytokines. Anti-miR-210 can inhibit the differentiation of Th1/Th17 cells and reduce the expressions of interleukin (IL)-17A and interferon-γ (INF-γ), thereby reducing inflammation. In conclusion, multiple miRs were involved in the occurrence and development of psoriasis by targeting the corresponding target genes.

In recent years, miRs have garnered significant attention as promising targets for the treatment of psoriasis. Given that psoriasis results from complex interactions between immune cells and keratinocytes, miR-based therapies have been developed to address this condition. Additionally, the skin is the most permeable organ, allowing for effective delivery of therapeutic agents through various administration routes. Consequently, miRs can be effectively delivered into the skin through local administration. One research group successfully designed a biomimetic recombinant high-density lipoprotein (rHDL) nanocarrier gel containing miR-210 antisense (NG-anti-miR-210) to significantly reduce miR-210 expression in skin lesions and splenic CD4 T cells of imiquimod (IMQ)-induced psoriasiform mouse models through topical administration. This innovative material ameliorated erythematous dermatitis, attenuated scaling and acanthosis, and inhibited skin inflammatory cell infiltration in IMQ-induced mice. Furthermore, the administration of NG-anti-miR-210 also decreased the proportion of Th1 and Th17 cells, as well as lowered interleukin (IL)-17A and interferon-γ (INF-γ) mRNA levels in cutaneous tissues and splenocytes of the mice (39). Therefore, the local inhibition of miR-210 by rHDL nanocarriers effectively alleviated psoriasis-like inflammation in mice, suggesting that “combination therapy” targeting the miR-210 pathway may be a potential treatment strategy for psoriasis. In sum, several miRs with varying expression levels, target molecules, and effects are implicated in psoriasis (Figure 1). Although great progress has been made in identifying potential miRs involved in psoriasis, further studies are necessary to apply miRs for diagnosis, treatment, and prognosis evaluation in psoriasis.

### 2.2 Skin wound

Skin wound healing is a complex physiological process that involves hemostasis, inflammation, angiogenesis, remodeling, and scarring (40, 41). MiRs play a crucial role in the process of cutaneous wound healing. Alterations in the expression of specific miR at different stages may be associated with abnormal wound healing (42–44). For instance, miR-132 has been shown to be substantially expressed in dermal fibroblasts after isolation from human skin wound tissue (45).

The cellular and molecular mechanisms that contribute to the delay in age-related cutaneous wound healing delaying remains unclear. Intradermal injection of the miR-21 plasmid around skin wounds improved healing and alleviated age-related skin wound defects. Up-regulation of miR-21 expression also accelerated wound healing in aged mice, suggesting that miR-21 may serve as a novel target during wound repair in aged mice (46). Phosphatase and tensin homolog (PTEN) is a known downstream target of miR-21 in various tumors. On one hand, the inhibition of PTEN promotes the wound healing process (47). On the other hand, miR-21 accelerates wound healing by increasing dendritic cells (DCs) through PTEN inhibition via the protein kinase B (Akt)/phosphatidylinositol-3 kinase (PI3K) signaling pathway (48). Recently, microvesicles (MVs) have emerged as an important medium for cell communication, capable of delivering genetic material to target cells. MV-derived miR-21 has been shown to regulate α-smooth muscle actin (α-SMA) and N-cadherin, facilitating the differentiation of fibroblasts into myofibroblasts and promoting fibroblast migration. MV-derived miR-21 upregulates the expression and secretion of IL-6 and IL-8, mediating inflammatory responses and enhancing immune responses. In addition, MV miR-21 downregulates PTEN and reversion-inducing cysteine-rich protein with Kazal motifs (RECK) protein expression, activates the MAPK/extracellular signal-regulated kinase (ERK) signaling pathway, and promotes the migration and differentiation of fibroblasts (40). This provides a foundation for the role of MV miR-21 in wound healing. PTEN has also been previously proven to promote cell proliferation and migration. However, whether PTEN is essential for MV miR-21-mediated fibrotic gene expression requires further investigation. Notably, miR-21 expressed in adipose-derived stem cells exosomes (AD-exos) has been demonstrated to play a role in wound healing, as well as in the proliferation and migration of keratinocytes. Overexpression of miR-21 suppresses the expression of transforming growth factor (TGF)-β1, while excess TGF-β1 exerts negative feedback on miR-21 (49). Thus, miR-21 is regulated by multiple upstream and downstream targets to accelerate skin wound healing. This offers a new perspective on the role of miR-21 in tissue repair, suggesting it could be a viable target for wound healing therapies.

Although the relationship between miR-19b and TGF-β1 remains unclear, it has been established that miR-19b derived from human adipose-derived mesenchymal stem cells (ADMSCs) improves cutaneous wound healing by targeting C-C motif chemokine ligand 1 (CCL1) via the TGF-β pathway (50). Additionally, the knockdown of apoptotic signal-regulating kinase 1 (ASK1) has been shown to reduce the expression of inflammatory factors *in vitro*. Specifically, miR-23b mediates ASK1 to inhibit inflammation, thereby promoting wound healing. These findings indicate that miR-23b serves as an effective therapeutic agent for wound healing by facilitating wound re-epithelialization, shortening the inflammatory response time, and accelerating keratinocyte migration (42). MiR-23b also negatively regulates tissue inhibitor of metalloproteinase-3 (TIMP-3). One study demonstrated after TGF-β1 stimulation in HaCaT cells, the abundance of miR-23b was positively correlated with both the concentration and duration of TGF-β1. Moreover, miR-23b promotes keratinocyte migration by downregulating TIMP-3 (51). Extracellular vesicles (EV)-encapsulated miR-106b exhibits inhibitory effects on the adhesion and viability of fibroblasts and keratinocytes. Conversely, the expression of two important mediators of angiogenesis, namely vascular endothelial growth factor (VEGF) and TGF-β1, was decreased following treatment with EV-encapsulated miR-106b, further suggesting that miR-106b may impede wound healing by inhibiting the angiogenesis process. Notably, there were no corresponding alterations in pro-inflammatory cytokine, indicating that inflammation itself may not be influenced by EV-encapsulated miR-106b in this skin wound healing model. These findings demonstrate that EV-encapsulated miR-106b could represent a promising strategy for regulating skin wound healing (52). Collectively, the varying effects on multiple miR expressions and the healing process mediated by TGF-β warrant further investigation.

In chronic wounds, biofilms can infect host tissues for extended periods. A research group has identified the underlying mechanisms by which biofilm-induced miR-146a and miR-106b affect host skin at the wound edge tissue. Zona occludens (ZO) proteins are ubiquitous scaffolding proteins that assemble multiprotein complexes on the cytoplasmic surface of the plasma membrane, linking transmembrane proteins to the filamentous cytoskeleton. MiR-146a and miR-106b silence ZO-1 and ZO-2, impairing tight junction function and resulting in compromised skin integrity. The 3’UTR regions of ZO-1 and ZO-2 are direct targets of miR-146a, while miR-106b targets either the exon or the 5’UTR region of the mRNA to inhibit gene expression. The findings demonstrate that topical delivery of miR-146a and miR-106b inhibitors to skin impaired by biofilm-infected wounds has the potential to restore barrier function and promote effective wound closure. This study reveals that biofilms may induce host skin miRs to impair skin function. It also lays the groundwork for intervention strategies aimed at inhibiting these miRs to restore skin barrier function (53).

Additionally, miR-212 knockdown alleviated beneficial effects of resveratrol on keratinocyte proliferation and migration, thereby inhibiting skin wound healing (54). Similarly, circular RNA protein kinase, DNA-activated, catalytic subunit (circ_PRKDC) impeded wound healing in diabetes by regulating keratinocyte proliferation and migration, which are crucial for skin wound healing. MiR-31 was identified as a target of circ_PRKDC, and the inhibition of miR-31 reversed the promoting effect on human epidermal keratinocyte (HEKa) migration following circ_PRKDC knockdown. In addition, miR-31 overexpression accelerated HEKa migration through fibrillin-1 (FBN1), suggesting that the knockdown of circ_PRKDC may accelerate wound healing by promoting keratinocyte migration through the miR-31/FBN1 axis (55). Research has confirmed that zinc finger E-box binding homeobox 1 (ZEB1) promotes skin wound healing. Moreover, the upregulation of ZEB1 resulted in the downregulation of miR-206 due to impaired binding to the miR-206 promoter. Overexpression of miR-206 or depletion of VEGFA counteracted ZEB1-induced proliferation, migration, and angiogenesis in human dermal microvascular endothelial cells (HDMEC), thereby delaying skin wound healing. In conclusion, ZEB1 enhances angiogenesis to promote skin wound healing by inhibiting miR-206 and increasing VEGFA expression (56). One study demonstrated that miR-21-5p and miR-125b-5p derived from umbilical cord blood mesenchymal stem cells (UCB-MSC)-derived exosomes inhibited TGF-β receptor type II (TGFBR2) and TGFBR1, respectively, thereby affecting the TGF-β1 signaling pathway and hindering myofibroblast differentiation. UCB-MSC-exosomes may represent a novel strategy to prevent scarring during wound healing in clinical settings (57). These findings deepen the understanding of the pathogenesis of cutaneous wound healing and provide potential therapeutic targets for enhancing skin wound healing.

Excessive collagen production and improper collagen deposition during wound healing can lead to cause skin scarring. MiRs are recognized as endogenous regulators in the formation of skin scars (58). A study demonstrated that the local injection of miR-29b lentiviral particles suppressed the expression of heat shock protein 47 (HSP47), blocked collagen synthesis, and inhibited angiogenesis, thereby alleviating scar formation. This study suggests that targeting the miR-29b/HSP47 pathway may provide an alternative approach to prevent or attenuate scar formation (59). In addition, miR-21-5p regulates cell migration via the PTEN/AKT signaling pathway. Electron beam (EB) irradiation inhibited autophagy in keloid fibroblasts by decreasing miR-21-5p levels. These findings indicate that EB irradiation modulates miR-21-5p, affecting autophagy, migration, and apoptosis in keloid fibroblasts, ultimately preventing local invasion and recurrence (60). Taken together, miR-21-5p has the potential to serve as a novel therapeutic target for keloid instead of EB irradiation.

### 2.3 Diabetic skin wound

Diabetes mellitus (DM) is a chronic metabolic disorder characterized by elevated blood glucose levels due to insulin imbalance or resistance. Clinical cutaneous symptoms often present as the earliest manifestations in patients with diabetes, increasing the risk of infections, hyperpigmentation, dermal thickening, spontaneous blister formation, and potentially life-threatening foot ulcers (61). A comprehensive investigation into the underlying molecular mechanisms that diabetic skin wound healing delaying may aid in mitigating complications associated with diabetic skin.

#### 2.3.1 Inflammation

The primary reasons for delayed wound healing in diabetes include a persistent inflammatory response, reduced expression of growth factor, and aggravated endothelial dysfunction (62, 63). Compared to streptozotocin (STZ)-induced diabetes of wild-type (WT) mice, corneal and skin wound healing was delayed, while neutrophil infiltration was increased around the skin wound in diabetic miR-146a knockout (KO) mice. This suggests that miR-146a deficiency impairs wound healing by increasing the inflammatory response in diabetic mice (64). These findings indicate that miR-146a could be a promising target for improving skin wound healing (65). Curcumin has been shown to promote diabetic wound healing despite of low bioavailability. One study has found that its synthetic analog, (2 E, 6 E) -2,6-bis (2- (trifluoromethyl) benzylidene) cyclohexanone (C66), increased miR-146a levels, downregulated the levels of phosphorylated NF-κB p65 subunit (p-p65) and interleukin-1 receptor-related kinase 1 (IRAK1), and inhibited the expression of inflammation-related cytokines in wounds of STZ-induced diabetic mice. C66 also counteracted high-glucose (HG)-induced NF-κB activation in HUVECs by upregulating miR-146a expression (66). In addition, miR-497 was found to reduce the expression of pro-inflammatory factors such as IL-1β, IL-6, and tumor necrosis factor-α (TNF-α) both *in vivo* and *in vitro*. The expression of miR-497 was significantly lower in hyperglycemic human dermal fibroblasts and WI-38 cells (67). These studies indicate that miR-146a and miR-497 are not only effective inflammatory markers but also potential targets for the treatment of diabetic skin wounds. However, earlier studies showed that miR-497 was downregulated in type 1 diabetes while upregulated in type 2 diabetes, respectively (68). These inconsistent results regarding miR-497 in diabetes may be attributed to difference in sample sources and the conditions of diabetic models.

#### 2.3.2 Angiogenesis

**FIGURE 2:**
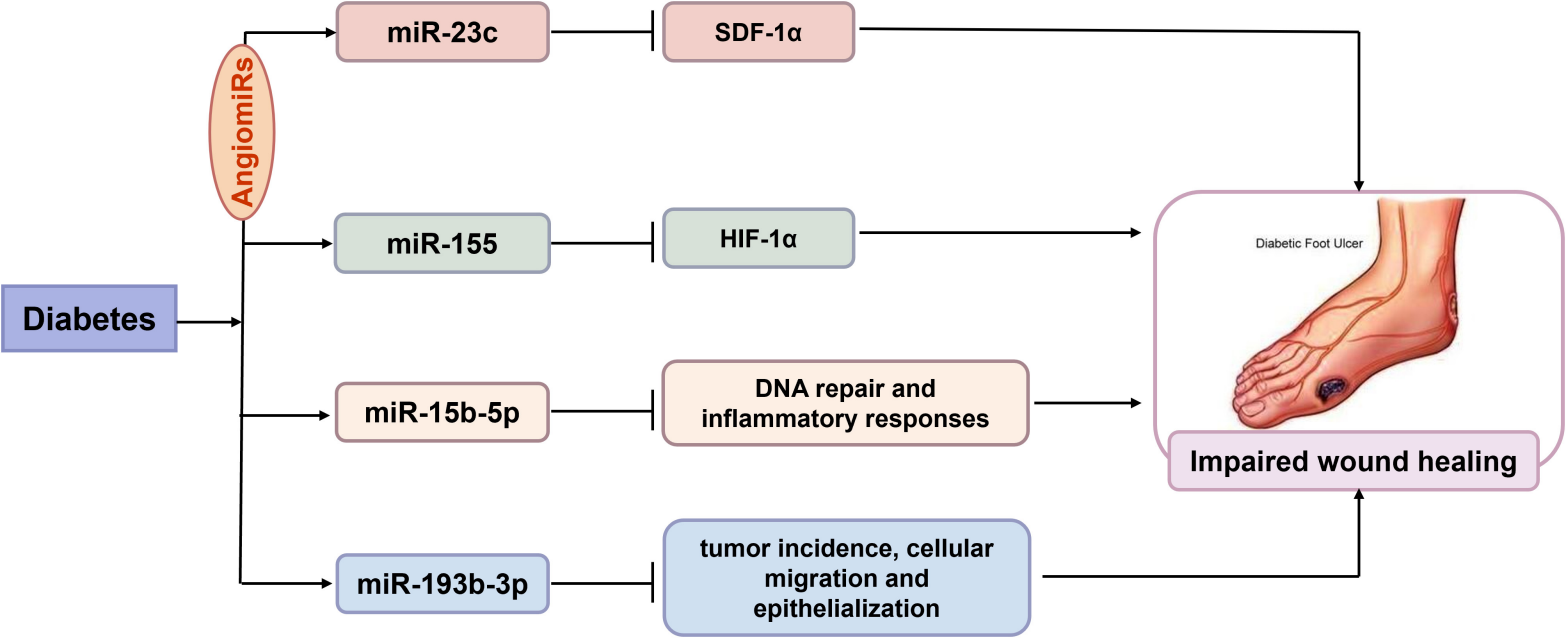
Possible involvement of microRNAs (miRs) in human diabetic skin wounds. There is growing evidence that miRs play a crucial role in regulating angiogenesis, called “AngiomiRs” including miR-23c and miR-155. MiR-23c negatively targeted stromal-derived factor-1α (SDF-1α) to exert an inhibitory effect on angiogenesis in diabetes. MiR-155 inhibited hypoxia-inducible factor 1-alpha (HIF-1α) expression to delay wound closure in diabetic wound tissue. In addition, miR-15b-5p suppressed DNA repair and inflammation in diabetic foot ulcers. And miR-193b-3p Cell migration and epithelialization acted as an inhibitor of cell migration and epithelialization in diabetic foot ulcers. Taken together, miR-23c, miR-155, miR-15b-5p and miR-193b-3p were involved in the impaired human diabetic wound healing by targeting respective target genes.

It has been reported that approximately 25% of diabetic patients suffer from diabetic foot ulcers (DFU), which are among the most common complications in patients with diabetic peripheral neuropathy, vascular disease, and foot deformities. The delayed healing of DFU is primarily attributed to impaired cellular activity, persistent inflammation, and enhanced oxidative stress associated with hyperglycemia (69). There is growing evidence that miRs play a crucial role in regulating angiogenesis, called “AngiomiRs” (70, 71). Delayed wound healing has been linked to abnormal production of stromal-derived factor-1α (SDF-1α), chronic inflammation, and reduced angiogenesis. MiR-23c has been found to be negatively associated with SDF-1α, exerting an inhibitory effect on angiogenesis by targeting this factor (72). Similarly, miR-92a-3p has also been identified as an effective miR that inhibits angiogenesis. MRG-110, a novel nucleic acid-modified inhibitor of miR-92a, upregulates the expression of integrin α5, a target gene of miR-92a with pro-angiogenic activity, thereby increasing angiogenesis and promoting epithelialization, which facilitates wound closure (73). In addition, local inhibition of miR-155 reduced macrophage and T-cell infiltration in wounds and suppressed tissue inflammation. Inhibition of miR-155 improved re-epithelialization and accelerated wound closure in diabetic wound tissue by increasing the expression of fibroblast growth factor 7 (FGF-7). These findings suggest that local inhibition of miR-155 reduces excessive inflammation and enhances tissue re-epithelialization and remodeling, which is beneficial for the healing of chronic DFU (74). A research team analyzed the expression of lncRNA cancer susceptibility candidate 2 (CASC2) in ulcer tissue from both human patients and mice. The results indicated that lncRNA CASC2 directly targeted miR-155, while hypoxia-inducible factor 1-alpha (HIF-1α) acted as a target gene of miR-155. Overexpression of miR-155 abolished the function of lncRNA CASC2, whereas inhibition of HIF-1α reversed the downregulation effects of miR-155 on fibroblasts. This study showed that overexpression of lncRNA CASC2 promoted wound healing through the miR-155/HIF-1α pathway in DFU (75). The research suggested that miR-155 is involved in the diabetic wound healing process in both animals and humans. Additionally, miR-15b-5p regulates several cellular processes, including DNA repair and inflammatory responses, by inhibiting downstream targets. Scientists constructed a human wound model to demonstrate that S. aureus-triggered miR-15b-5p suppress inflammation and DNA repair-related genes, resulting in the accumulation of DNA double-strand breaks (DSB) that subsequently facilitate a persistent, unresolved inflammatory state in DFU. That is to say, miR-15b-5p may serve as a master regulator and a potential therapeutic target and/or a biomarker in DFU (76). It was also noted that plasma levels of miR-193b-3p were elevated in patients with diabetes, although its specific role in DFU remained unknown (77). Notably, recent studies have demonstrated that miR-193b-3p is highly expressed and inhibits wound closure in human organotypic wound models, while knockdown of miR-193b-3p accelerates wound reepithelialization. Mechanistically, miR-193b-3p mediates anti-migratory activity by disrupting stress fiber formation and reducing the activity of GTPase RhoA. It suggested that miR-193b-3p acted as an inhibitor of cell migration and epithelialization in DFU (78). In summary, miR-23c, miR-155, miR-15b-5p and miR-193b-3p are implicated in the impaired diabetic wound healing by targeting specific target genes in human (Figure 2). The aforementioned studies on miRs indicated that specific miR inhibitors may be beneficial in promoting diabetic skin wound healing by exerting anti-inflammatory effects and promoting angiogenesis.

### 2.4 Burn

Slow wound healing, along with susceptibility to infection and excessive scar formation, remains a significant challenge in burn treatment. Burn wound healing is a multi-step process that involves various cells and biochemical factors (79). Insights into local tissue changes in the skin during the early stages of burns can help identify additional biomarkers for burn treatment (80). Similar to the critical role of AngiomiRs in diabetic skin wound healing, as above mentioned, AngiomiRs are also important in the process of burn wound healing. MiR-126 is an endothelial-specific miR associated with angiogenesis and vascular integrity. Expression levels of miR-126 were found to be elevated, while HOX transcript antisense intergenic RNA (HOTAIR) and Sciellin (SCEL) were downregulated in burn tissue and heat stress-exposed HUVECs. Further research demonstrated that miR-126 promoted endothelial cell migration, proliferation, and angiogenesis while inhibited apoptosis. In contrast, both HOTAIR and SCEL exhibited effects opposite to those of miR-126 in HUVECs. Studies *in vivo* showed that miR-126 also promoted burn wound healing by mediating angiogenesis (81). In addition, burns trigger a systemic response characterized by increased vascular permeability. A group of researchers observed that miR-451 expression was elevated in endothelial cells within a rat model, which inhibited angiogenesis and increased endothelial cells permeability (82). Although validation from experiments *in vivo* is still needed, several miRs may emerge as novel targets for burn therapy.

In human dermal fibroblasts and keratinocytes, miR-486 and miR-663 directly target Bcl-2-like protein 14 (BCL2L14), acting as an apoptotic activator. The overexpression of miR-486 or miR-663 increases keratinocyte proliferation while inhibiting human skin fibroblast apoptosis. Notably, lidocaine is currently widely used in clinical settings for pain relief. Studies have shown that lidocaine promotes post-burn skin healing by upregulating the expression of miR-486 and miR-663, which may provide a new theoretical basis for its use in post-burn skin treatment (83). Another research team investigated the effects of iPSCs-derived microvesicles (iPSCs-MVs) on deep second-degree burn wounds of a mouse model. They found that iPSCs-MVs activated the p38/MAPK pathway and promoted keratinocyte migration by targeting desmoglein 3 (Dsg3) via miR-16-5p. Moreover, the topical application of miR-16-5p facilitated keratinocyte migration, thereby promoting re-epithelialization of burn wounds (84). MiR-16-5p could represent a promising new therapeutic option for deep second-degree burn wounds. However, miRs have multiple target genes, and miR-16-5p can regulate keratinocyte migration through various pathways. While the current study has only clarified the role of Dsg3 in this process, other underlying mechanisms remain to be elucidated.

Recently, miR-135-5p has been linked to prognosis following skin transplantation in burn patients. The proviral integration site of murine 2 (PIM2) has been identified as a common target in anti-apoptotic pathways that increase the survival of skin grafts in individuals with severe burns. PIM2 has also been recognized as a virtual target of miR-135-5p, which exhibits a negative regulatory relationship with miR-135-5p. In summary, miR-135-5p negatively impacts with cell viability and apoptosis (85). In addition, miR-506-3p has been characterized as either a tumor suppressor or an oncogene in fibroblasts derived from various tumors. A study revealed that miR-506-3p regulates autophagy and proliferation in post-burn skin fibroblasts through post-transcriptionally suppressing Beclin-1 expression (58). This study provides a promising approach for skin healing treatments following burn. However, the safety and bioavailability of applying these techniques in clinical applications still remain to be explored.

### 2.5 Systemic sclerosis

Systemic sclerosis is a chronic autoimmune disease characterized by immune disorders, vascular lesions, and fibrosis of the skin and internal organs (86). It is well-known that skin fibrosis serves as an important indicator of various diseases, profoundly affecting the patient’s physical condition and life quality (87). Numerous studies have confirmed that miRs are potential regulators of disrupted signaling pathways involved in fibrosis (88). Many newly miRs are now associated with both organ-specific and systemic fibrosis. Recently, the targets of these altered miRs have been validated, and new biochemical pathways have been defined (89). It is worth mentioning that miRs have been implicated in vascular injury, immune activation, and fibroblast activation. Therefore, miRs may serve as potential biomarkers in the progression of SSc (90, 91). One study identified 21 miRs and 2698 miRs that were differentially expressed in SSc (92, 93). Detailly, 17 miRs and 33 target miRs (55 miR-mRNA pairs) were involved in Wnt signaling, Toll-like receptor and TGF-β pathways. MiR-21, miR-31, miR-130b, miR-146b, and miR-34a expression were elevated, while miR-145 expression was suppressed in SSc skin tissues, fibroblasts, and endothelial cells stimulated with serum from SSc patients (94). Although further investigation into the underlying mechanisms is still required, this study more or less illustrated that miRs play a crucial role in SSc.

Recently, a research team found that miR-125b was downregulated in skin tissues, particularly in human dermal fibroblasts from SSc patients. Mechanistically, the downregulation of miR-125b increased apoptosis, promoted dermal fibroblast proliferation, and increased α-SMA expression, suggesting a protective effect against the progression of skin fibrosis in SSc. This finding may provide a novel strategy for the treatment (87). Dickkopf-1 (DKK-1) serves as a negative regulator in SSc fibrosis, and is found to be decreased in SSc skin tissue and fibroblasts, rather that in blood. DKK-1 has been identified as a direct target of miR-33a-3p, which epigenetically downregulates the expression of DKK-1 in tissues and cells from SSc. Consequently, restoring DKK-1 levels through epigenetic modulation of miR-33a-3p may represent a promising approach for SSc treatment (95). A variety of miRs post-transcriptionally regulate gene expression by binding to the 3’UTR of their target genes, which can result in multiple disorders in SSc. For instance, exogenous miR-5196 has been shown to bind to the 3’UTR of the fos-related antigen 2 (fra2) gene and reverse monocyte fibrosis. The application of 3-Deazaneplanocin A (DZNep) and toll-like receptor 8 agonists promoted the production of profibrotic factors, including reactive oxygen species (ROS), TIMP-1 and IL-8 in monocytes from patients with SSc. These findings may be attributed to the downregulation of miR-5196 in monocytes from SSc patients. In addition, the expression of Fra2 and TIMP-1 was reduced following the exogenous transfection of miR-5196, indicating its potential as a target for fibrogenesis in SSc (91). In sum, miRs provided new insights for the diagnosis and treatment of early-stage SSc.

Some researchers have examined various miRs in skin biopsies from patients with SSc and healthy controls. They confirmed that miR-21 and miR-29a regulate collagen production in opposing manners. The TGF-β-induced fibrotic response in dermal fibroblasts can be attenuated by enhancing miR-29a expression or reducing miR-21 activity. Therefore, maintaining the balance between miR-21 and miR-29a presents a promising strategy for treating SSc and other fibrotic diseases characterized by abnormal collagen expression (88). However, the underlying molecular mechanisms require further exploration in the future. In a bleomycin-induced mouse model, the local injection of chemokines (C-X-C motif) ligand (CXCL) 17 significantly attenuated skin fibrosis in mice. In the skin tissue of patients with SSc, CXCL17 expression was detected to be considerably lower than that in healthy controls, while CXCL17 expression were elevated in the serum of patients with SSc. The low expression of CXCL17 in the skin tissue of SSc patients influenced the accumulation of type I collagen. Notably, CXCLs have been identified as playing a role in the progression of SSc. The proposed mechanism suggests that CXCL17 post-transcriptionally regulates the expression of type I collagen through miR-29 and matrix metalloproteinase 1 (MMP1) (96). Therefore, an in-depth investigation of the detailed mechanism of miR-29-mediated regulation of collagen expression may provide a novel strategy for the treatment of SSc.

### 2.6 Skin tumors

#### 2.6.1 Melanoma

Melanoma is a malignant skin cancer characterized by a high mortality rate, and current treatment outcomes are often suboptimal. In 2006, a study first reported the presence of miRs existed in melanoma cells. Since then, an increasing number of researchers have investigated miR profiles in melanoma to identify novel biomarkers (97, 98). Recent studies have revealed that miRs play essential roles as regulators in the angiogenesis of melanoma (99–101). Specific miR characteristics have demonstrated significant diagnostic, prognostic, and predictive value, with line-specific and immune-related miRs frequently identified as important markers. Currently, the potential of circulating miRs as biomarkers for melanoma has been explored in the literature, highlighting the necessary advancements to translate miR research into therapeutic applications (102). Several melanoma-associated miRs have been identified as functioning either upstream or downstream of known melanoma oncogenes. MiR-23b was found to be downregulated in melanoma tissue and correlated with reduced patient survival. Consequently, the upregulation of miR-23b effectively impaired cell viability and colony formation, inhibited angiogenesis, and accelerated apoptosis in SK-MEL-28 cells (103). This suggests that miR-23b may serve as a potential preventive factor in melanoma.

In recent years, several research has uncovered the molecular mechanisms underlying malignant melanoma and identified various potential therapeutic targets (104–106). MiR-214 analogs have been shown to promote survival and migration of melanoma cell. Notably, the expression of cell adhesion molecule 1 (CADM1) was decreased in melanoma cells. However, miR-214 expression was considerably enhanced during the progression of melanoma. Specifically, miR-214 promoted epithelial-mesenchymal transition (EMT) by downregulating CADM1, while miR-214 inhibitor effectively blocked the EMT process (107). In addition, some studies have reported that miR-182 expression in malignant melanoma tissues is much higher than that in surrounding tissues. The downregulation of miR-182 expression prevented malignant melanoma cells from proliferating, whereas overexpression of miR-182 promoted their growth. In human malignant melanoma tissues, the reversion-inducing cysteine-rich protein with Kazal motifs (RECK) was downregulated. Moreover, low levels of miR-182 promoted RECK expression in malignant melanoma cells. MiR-182 regulated RECK expression and inhibited the proliferation of malignant melanoma cells, thereby providing a novel target for molecular therapy in the treatment of malignant melanoma (108). In addition to miR-214/CADM1 and miR-182/RECK, recent studies have demonstrated that the exosomal miR-29c-3p derived from M1 macrophages inhibits the invasiveness of melanoma cells through ectonucleotide pyrophosphatase/phosphodiesterase 2 (ENPP2) (109). Meanwhile, miR-650 facilitated melanoma metastasis by targeting the inhibitor of growth family member 4 (ING4). Additionally, extracellular vesicles derived from melanoma cells stimulated cancer-associated fibroblasts through the miR-92b-3p-mediated downregulation of PTEN (110, 111). These findings established a multi-miRNA regulatory axis that is crucial for the progression of melanoma. Therefore, miRs may serve as promising molecular targets for melanoma treatment.

Previous studies have demonstrated that miR-targeted therapy can influence melanoma and increase sensitivity to both conventional and immunotherapeutic approaches. Three miRs, specifically miR-495-3p, miR-376c-3p, and miR-6730-3p, were shown to be enriched in exosomes and microvesicle fractions in a P2X purinoceptor 7 (P2 × 7)-dependent manner. These three miRs promoted the proliferation and migration of melanoma cells, while antagonism of P2 × 7 alleviated their vesicle release. This suggests that the pro-metastatic activity of the P2 × 7 receptor may be mediated through exosomes/microvesicles and miR (112). Additionally, another study indicated that overexpression of miR-107 reduced the migration, proliferation, and invasion of melanoma cells. The overexpression of POU domain, class 3, transcription factor 2 (POU3F2), a downstream target of miR-107, antagonized miR-107-mediated suppression of melanoma cells, highlighting miR-107 as a novel tumor suppressor during melanoma metastasis (113). Therefore, these miRs represent potential biomarkers for melanoma, providing new strategies for the development of personalized treatment approaches.

#### 2.6.2 Squamous cell carcinoma (SCC)

Squamous cell carcinoma is a lethal malignancy characterized by a high propensity for metastasis (114). Studies have demonstrated that elevated expression of tumor necrosis factor-α-induced protein 8 (TNFAIP8) is associated with the progression of SCC. Induction of TNFAIP8 expression in SCC cell lines promotes cell growth. Conversely, silencing of TNFAIP8 diminishes cell survival and reduces cell migration. Moreover, the study also confirmed that miR-205-5p targets the 3’UTR of TNFAIP8, leading to the inhibition of TNFAIP8 expression. Accordingly, miR-205-5p downregulates TNFAIP8-mediated autophagy, increases sensitivity to vemurafenib, and induces apoptosis in the cells. Therefore, miR-205-5p acts as a tumor suppressor in SCC by targeting TNFAIP8 (115). In addition, one research team disrupted the expression of the tumor suppressor Grainyhead-like 3 (GRHL3), leading to a loss of PTEN and the activation of the PI3K/mammalian target of rapamycin (mTOR) signaling pathway. This disruption promotes the development of aggressive SCC in both mouse and human skin. Experimental results indicated that resistant SCC exhibited increased miR-21 expression, while antagonists of miR-21 restored GRHL3 and PTEN expression levels (116). Recently, a team conducted a comprehensive investigation into the expression levels of miR-34 family members in patients with SCC. They found that the levels of miR-34a and miR-34b/c were significantly decreased in these patients. Moreover, both miR-34a and miR-34b/c suppressed the proliferation, migration and invasion of SCC cells via the Notch1 signaling pathway, with miR-34b/c exhibiting a stronger inhibitory effect than miR-34a. Furthermore, miR-34a showed a significant association with CD44 levels in the patients. The knockdown of CD44 significantly reduced the miR-34a-mediated inhibition of cell migration and invasion. This study confirmed that miR-34 family members act as negative regulators of SCC, with their inhibitory effects potentially mediated by multiple complex signaling pathways (117). Additionally, in healthy epithelia, follistatin-like 1 (FSTL1), a pro-metastatic glycoprotein, experiences mRNA destabilization by binding of KH-type splicing regulatory protein (KSRP), which processes it into primary miR encoding miR-198. However, the downregulation of KSRP in SCC terminates miR-198 processing, thereby facilitating FSTL1 translation. The deletion of miR-198 leads to the aberrant expression of the pro-migratory targets. Subsequently, pro-invasion proteins, in conjunction with FSTL1, enhance SCC invasion and metastasis (118). Therefore, miR-198 presents a potential biomarker candidate for SCC prognosis. A suitable FSTL1 inhibitor, combined with a compound to restore the expression of the tumor suppressor miR-198, may significantly limit metastatic spread in SCC.

#### 2.6.3 Basal cell carcinoma (BCC)

Few studies have investigated the differential expression of miRs in BCC, and the regulatory roles of miRs in BCC development remain unclear. MiR-451a has been identified as a tumor suppressor in cutaneous BCC. By assessing miR-451a levels in human BCC tissues and in an inducible BCC mouse model, researchers found that miR-451a was significantly reduced. The overexpression of miR-451a in tumor cells markedly inhibited cell growth by inducing G1 cell cycle arrest, while the inhibition of miR-451a in primary cells promoted cell growth and colony-forming ability. Further studies confirmed T-box 1 (TBX1) as a downstream target of miR-451a, indicating that miR-451a/TBX1 axis plays a critical role in BCC tumorigenesis. In subsequent stage, it will be essential to verify the therapeutic effects of the topical application of miR-451a, which will provide a comprehensive understanding of its clinical efficacy of BCC treatment (119). Another team collected tissue samples from 20 patients with BCC and 20 healthy controls (HC) to compare the expression of miR-18a in these samples. They found that miR-18a exerts oncogenic effects through the Akt/mTOR/Beclin 1/protein light chain 3 (LC3) pathway, and the antitumor effects of miR-18a inhibitors may be a promising approach for the treatment of BCC (120).

Taken together, the studies mentioned above reveal the regulatory effects and fundamental biological roles of miRs in skin tumors. This understanding will contribute to elucidating the molecular pathogenesis of melanoma, SCC and BCC.

### 2.7 Other dermatological diseases

Recessive dystrophic epidermolysis bullosa is a skin fragility disorder caused by mutations in the COL7A1 gene, which encodes type VII collagen. This condition is characterized by persistent blistering, chronic wounds with significant inflammation and fibrosis. Inhibition of miR-145p-5p in RDEB skin fibroblasts significantly increased the expression of the transcriptional repressor Krüppel-like factor 4, which regulates contractile proteins, inhibited the fibrosis inducer Jagged1, and ultimately reduced the levels of the contractile markers α-SMA and transgelin (121). These data highlighted the profibrotic role of miR-145-5p and associated regulatory networks in RDEB, shedding light on novel pathomechanisms and potential therapeutic targets for future interventions.

Systemic lupus erythematosus is an autoimmune illness with an unknown cause and pathogenic mechanism. The persistent autoimmune response associated with SLE can damage tissues or organs, leading to lesions or relevant symptoms. Recent studies have highlighted the crucial role of miRs in regulating both innate and adaptive immune responses (122–124). Subacute cutaneous lupus erythematosus (SCLE) and discoid lupus erythematosus (DLE) are two common forms of the disease that present with skin lesions in 80 percent to 90 percent of patients. Specific overexpression of miR-31 activated NF-κB pathway, leading to apoptosis in keratinocytes and the production of inflammatory cytokines that promote the recruitment of neutrophil and monocyte to sites of inflammation. Stimulation with IL-1α and TGF-β1 elevated the expression of miR-485-3p in peripheral blood monocytes from patients with DLE and triggered T cell activation. Furthermore, overexpression of miR-485-3p promoted fibrosis in dermal fibroblasts by targeting peroxisome proliferator-activated receptor-gamma coactivator-1α (PGC-1α) (125). This study demonstrates that overexpression of miR-31 and miR-485-p regulates the production of inflammatory factors, recruits neutrophils and monocytes to the skin, and induces skin inflammation associated with DLE, thereby providing new insights for clinical treatment of DLE.

It is worth noting that miRs also play a significant role in many other skin diseases, including photoaging, alopecia, pigmentation disorders (such as chloasma, vitiligo, and albinism), acne, dermatitis, and urticaria (12–17). These studies demonstrate that miRs have regulatory effects on various dermatological diseases.

## 3 Clinical study and novel applications of miRs in dermatological diseases

Currently, some scientists are investigating miRs for clinical research in dermatological diseases. A research team selected 75 patients with significant skin damage from the confirmed endemic areas of coal-burning arsenism as study subjects. Research indicated that Ginkgo biloba downregulated intracellular miR-155-5p and improved arsenic-induced immune dysfunction, thereby reducing the expression of biomarkers associated with arsenic-induced skin damage (126). As mentioned above, certain miRs can regulate angiogenesis, with miR-424 functioning as a tumor suppressor gene. Specifically, miR-424 modulates VEGF and basic fibroblast growth factor (bFGF) signaling in HUVECs by targeting VEGF, VEGFR2, and fibroblast growth factor receptor 1 (FGFR1), thereby inhibiting endothelial cell proliferation, migration, tube formation, and angiogenesis. Conversely, miR-424 promotes angiogenesis in hypoxic endothelial cells, suggesting that its effects may be context-dependent. Notably, a clinical study found low expression levels of miR-424 in infantile skin hemangioma tissue. Furthermore, miR-424 can inhibit the bFGF/FGFR1 pathway and suppress ERK1/2 phosphorylation, thereby attenuating cell proliferation, migration, and tube formation capacity, ultimately delaying the development of cutaneous hemangiomas in infants (127). A project evaluated angio-miRs (miR-92a, miR-126-3p, miR-221, miR-222) and inflamma-miRs (miR-21-5p, miR-146a-5p) in psoriasis patients undergoing adalimumab treatment. The study confirmed that reductions of miR-146a levels were associated with improvements in the Psoriasis Area and Severity Index (PASI), suggesting that the inhibition of miR-146a may help alleviate psoriasis (128). Nevertheless, larger-scale clinical studies are indispensable to determine and fully validate the clinical role of miRs in dermatological diseases.

**FIGURE 3:**
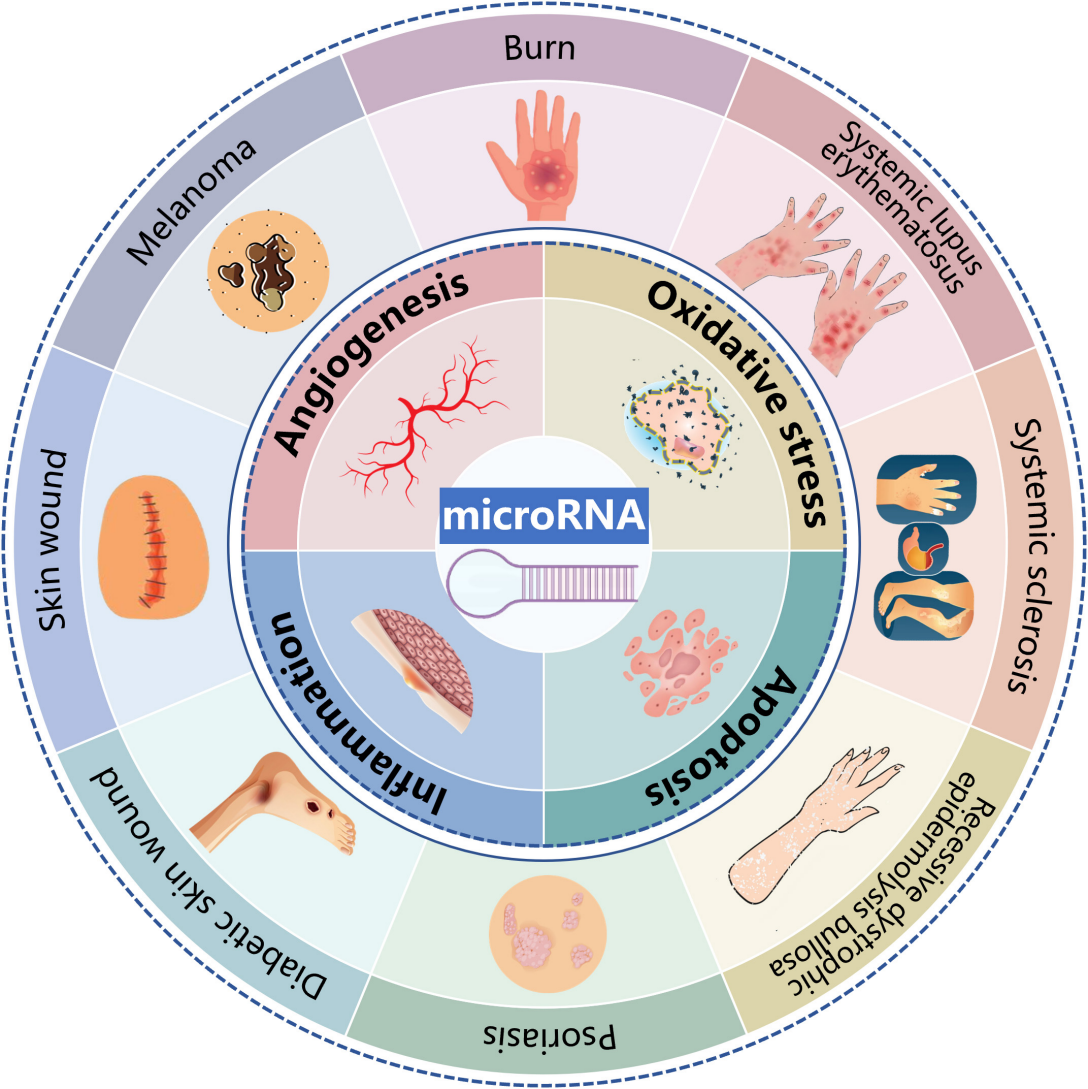
Potential effects and mechanisms of microRNAs (miRs) in dermatological diseases. MiRs influences a wide range of pathophysiological processes of dermatological diseases including psoriasis, skin wound, diabetic skin wound, burn, systemic sclerosis, skin tumors, recessive dystrophic epidermolysis bullosa, and systemic lupus erythematosus. In terms of mechanism, regulation on oxidative stress, inflammation, apoptosis and angiogenesis might be responsible for the distinct roles of miRs in skin.

Over the past few decades, significant progress has been made in understanding the potential effects of miRs on various dermatological diseases (129–131). The regulation of oxidative stress, inflammation, apoptosis, and angiogenesis may account for the distinct roles of miRs in skin (Figure 3). However, the precise mechanisms by which miRs influence skin diseases remain to be fully elucidated. With the rapid advancements in materials science, the integration of miRs with novel materials presents new opportunities for treating skin diseases. A research group has developed a miR-21-mimicking nanocarrier system utilizing facial amphiphilic bile acid-conjugated polyethyleneimines (BA-PEI) for the intracellular and transdermal delivery. These miRs nanocarrier systems have been shown to enhance the speed and quality of wound healing, promote collagen synthesis, and accelerate wound re-epithelialization (132).

Synovial mesenchymal stem cells (SMSCs) can enhance fibroblasts’ proliferation. However, their effect on promoting angiogenesis is not as pronounced. Recent studies have reported that the overexpression of miR-126-3p can transfer the angiogenic capacity of endothelial progenitor cells to SMSCs. Exosomes derived from SMSCs overexpressing miR-126-3p (SMSC-126-Exos) activated human dermal fibroblasts and dermal microvascular endothelial cells (HMEC-1) in a dose-dependent manner. The gene overexpression in these modified cells allowed SMSC-126-Exos to serve as an efficient drug delivery system, presenting limitless possibilities for future therapies targeting skin wounds (133). Additionally, wound healing can be facilitated by both magnetic nanoparticles (MNPs) and exosomes produced from bone mesenchymal stem cells (BMSC-Exos) (134). BMSCs will produce novel exosomes (mag-BMSC-Exos) in response to the stimulation of MNPs and a static magnetic field (SMF). MiR-21 is a blood vessel-specific molecule that regulates angiogenesis and fibrosis. Notably, miR-21-5p has been found to be highly enriched in mag-BMSC-Exo, enabling it to be delivered into resident HUVECs and human skin fibroblasts. This delivery stimulates their regenerative response, thereby accelerating skin wound repair and regeneration. The upregulation of miR-21-5p may improve wound healing through the action of mag-BMSC-Exos (135). Therefore, the biologically active miR-21-5p can serve as a carrier for delivering therapeutic agents to recipient cells, providing a promising strategy for clinically promoting wound healing.

Multiple previous studies have shown that increased inflammation and decreased expression of miR-146a delay diabetic wound healing. To address this issue, a research team coupled miR-146a with cerium oxide nanoparticles (CNP), targeting ROS and inflammation. Administration of CNP-miR-146a at a dose of 100 ng shortened wound healing time, reduced inflammation, and increased angiogenesis, thereby promoting diabetic wound healing without compromising the biomechanical properties of the healed skin (136). In addition, anofilaments composed of silk fibroin successfully carried CNP-miR-146a and improve the biological properties of diabetic skin. This approach promoted the healing of diabetic wounds by synergistically reducing oxidative stress, inhibiting pro-inflammatory gene signaling, and promoting fibrosis (137). This nanotechnology-based therapy shows promising potential for future applications.

## 4 Pleiotropic roles of miRs across dermatological diseases

It is noteworthy that the same miR can exert divergent, and even opposing, effects in different dermatological conditions due to cell-type specificity, micro-environmental cues, and disease-specific target gene networks. This context-dependent functionality highlights the complexity of miR regulation in skin pathophysiology. Below, we summarize the pleiotropic roles of miR-21, miR-155, and miR-166a across various dermatological diseases (Table 1).

**TABLE 1:** The levels, target genes, primary functions, target cells and mechanisms of pleiotropic microRNAs (miRs).

MiRs	Disease	Levels	Target genes	Primary functions	Target cells	Mechanisms	References
miR-21	Psoriasis	↑	CD11b (ITGAM)	Disrupt macrophage function; Promoted keratinocyte proliferation	Keratinocytes, Macrophages	Immune dysregulation	(32)
	Skin wound	↑	PTEN/PI3K-AKT	Enhanced fibroblast migration; Accelerated re-epithelialization	Dermal Fibroblasts	Pro-repair ECM remodeling	(48)
	Melanoma	↑	PTEN/RECK (via exosomes)	Promoted metastasis; Angiogenesis	Tumor cells, Stroma	Exosome-mediated tumor-stroma crosstalk	(108)
	Systemic sclerosis	↑	miR-29a/TGF-β	Drived collagen deposition	Dermal Fibroblasts	Fibrotic ECM stabilization	(88)
miR-155	Diabetic foot ulcers	↑	FGF-7/HIF-1α	Impaired re-epithelialization	Fibroblasts, Endothelial cells	Metabolic memory impairment	(75)
	Arsenic dermatopathy	↑	Immune regulators	Exacerbated immune dysfunction	Lymphocytes	Chemical toxicity response	(126)
miR-146a	Diabetic skin wound	↓	IRAK1/NF-κB	Failed to suppress inflammation	Macrophages, Keratinocytes	Hyperglycemia-induced signaling dysregulation	(66)
	Skin wound	↑	ZO-1, ZO-1	Restored barrier function	Keratinocytes	Silencing of tight junction proteins by biofilm infection	(53)

## 5 Conclusion

In recent years, miRs have been reported to be implicated in various pathophysiological processes of dermatological diseases, including psoriasis, skin wound, diabetic skin wound, burn, SSc, skin tumors (melanoma, SCC and BCC), RDEB, and SLE. Mechanistically, the regulation of oxidative stress, inflammation, apoptosis, and angiogenesis may account for the distinct roles of miRs in skin (Figure 3 and Table 2). A deeper understanding of different miRs and their related regulatory targets is essential for elucidating the pathophysiology of numerous skin diseases. Moreover, the combination of miRs with novel materials or compounds may offer innovative approaches for the treatment of skin diseases. However, further research is necessary to facilitate the translation into clinical applications for dermatological diseases.

**TABLE 2:** The levels, targets and effects of microRNAs (miRs) in dermatological diseases.

Diseases	MiRs	Levels	Targets molecules	Effects	References
Psoriasis	miR-215-5p	↓	EGFR	MiR-215-5p overexpression inhibited HaCaT cell proliferation and diminished psoriasis-like inflammation.	(27)
	miR-21	↑	caspase -8, apaf-1	MEG3/miR-21 axis contributed to the proliferation and apoptosis of psoriatic keratinocytes.	(31)
			CD11b	Upregulation of miR-21 expression induced Mac-1 function disturbance, innate immune cell abnormality, and the cytokines secretion excess in psoriasis pathogenesis.	(32)
	miR-744-3p	↑	KLLN	Up-regulated miR-744-3p in psoriasis regulated the proliferation and differentiation of keratinocytes by targeting KLLN.	(34)
	miR-146b, miR-10b	↑	ACKR2	Bound directly to the 3’-UTR of ACKR2, then suppressed ACKR2 transcription and protein expression in keratinocytes and lymphoid endothelium cells, respectively.	(35)
	miR-146a-5p, miR-21-5p, miR-122-5p	↑	MAPK, JAK, STAT, NF-κB	Acitretin inhibited inflammatory response and keratinocyte proliferation to attenuated the development of psoriasis via reducing miRs expression.	(36)
	miR-31	↑	STAT3, p53	Overexpression of miR-31 led to STAT3 upregulation and p53 enhancement, and induced keratinocytes hyperproliferation.	(37)
	miR-17-3p	↑	CTR9	MiR-17-3p promoted keratinocyte proliferation and pro-inflammatory cytokine secretion.	(38)
	miR-210	↑	IL-17A, INF-γ	NG-anti-miR210 ameliorated erythematous dermatitis, attenuated scaling and acanthosis, and inhibited skin inflammatory cell infiltration.	(39)
Skin wound	miR-21	↑	–	Intradermal injection of miR-21 plasmid around skin wounds improved healing and alleviating age-related skin wound defects.	(46)
			PTEN	MiR-21 accelerated wound healing by increasing DCs through PTEN inhibition via Akt/PI3K signal pathway.	(47)
			α-SMA, N-cadherin, MAPK, ERK	MV miR-21promoted the migration and differentiation of fibroblasts.	(40)
	miR-19b	↑	CCL1	Human ADMSCs-derived miR-19b improved cutaneous wound healing by targeting CCL1 via TGF-β pathway.	(50)
	miR-23b	↑	ASK1	MiR-23b mediated ASK1 to inhibit inflammation, thereby promoting wound healing.	(42)
			TIMP3	MiR-23b promoted keratinocyte migration by downregulating TIMP3.	(50)
	miR-106b	↓	VEGF, TGF-β1	MiR-106b delayed wound healing by inhibiting the angiogenesis process.	(52)
	miR-146a, miR-106b	↓	-	Topical delivery of miR-146a and miR-106b inhibitors restored barrier function and promoted wound closure.	(53)
	miR-212	↑	-	MiR-212 knockdown alleviated the improvement effects of resveratrol in keratinocyte proliferation and migration, thereby inhibiting skin wound healing.	(54)
	miR-31	↑	FBN1	MiR-31 overexpression accelerated HEKa migration through FBN1.	(55)
	miR-206	↓	VEGFA	ZEB1 enhanced angiogenesis to promote skin wound healing by inhibiting miR-206 and increasing VEGFA expression.	(56)
	miR-21-5p, miR-125b-5p	↑	TGFBR2, TGFBR1	MiR-21-5p and miR-125b-5p inhibited TGFBR2 and TGFBR1, respectively, thereby affecting the TGF-β1 signaling pathway to hinder myofibroblast differentiation.	(57)
	miR-29b	↑	HSP47	Local injection of miR-29b lentiviral particles suppressed the expression of HSP47, blocked collagen synthesis and inhibited angiogenesis, thereby alleviating scar formation.	(59)
	miR-21-5p	↑	PTEN/AKT	EB irradiation regulated miR-21-5p to affect autophagy, migration, and apoptosis of keloid fibroblasts, and finally prevented local invasion and recurrence.	(60)
Diabetic skin wound	miR-146a	↑	–	MiR-146a deficiency slowed wound healing by increasing the inflammatory response in diabetic mice.	(64)
			p-p65, IRAK1, NF-κB	C66 increased miR-146a levels, down-regulated the levels of p-p65 and IRAK1, and inhibited inflammation-related cytokine expression.	(66)
	miR-497	↓, ↑	IL-1β, IL-6, TNF-α	MiR-497 reduced the expression of pro-inflammatory factors *in vivo* and *in vitro*.	(67)
	miR-23c	↑	SDF-1α	MiR-23c exerted an inhibitory effect on angiogenesis by targeting SDF-1α.	(72)
	miR-92a-3p	↑	Integrin α5	MiR-92a inhibitor up-regulated the expression of integrin α5 to increase angiogenesis, promote epithelialization, thereby facilitating wound closure.	(73)
	miR-155	↑	FGF-7	Local inhibition of miR-155 reduced macrophage and T-cell infiltration in wounds and suppressed tissue inflammation.	(74)
		–	HIF-1α	Overexpression of lncRNA CASC2 promoted wound healing through miR-155/HIF-1α in DFU	(75)
	miR-15b-5p	–	–	MiR-15b-5p suppressed inflammation and DNA repair-related genes, leading to the accumulation of DNA DSB.	(76)
	miR-193b-3p	↑	–	MiR-193b-3p mediated anti-migratory activity by disrupting stress fiber formation and reducing the activity of GTPase RhoA.	(78)
Burn	miR-126	↑	–	MiR-126 promoted endothelial cell migration, proliferation, and angiogenesis but inhibited apoptosis.	(81)
	miR-451	↑	–	MiR-451 inhibited angiogenesis and increased endothelial cells permeability.	(82)
	miR-486, miR-663	↑	BCL2L14	Overexpression of miR-486 or miR-663 increased keratinocyte proliferation while inhibited human skin fibroblast apoptosis.	(83)
	miR-16-5p	–	Dsg3	IPSCs-MVs activated the p38/MAPK pathway and promoted keratinocyte migration by targeting Dsg3 via miR-16-5p.	(84)
	miR-135-5p	–	PIM2	MiR-135-5p interfered with cell viability and apoptosis.	(85)
	miR-506-3p	↑	Beclin-1	MiR-506-3p regulated autophagy and proliferation in post-burn skin fibroblasts through post-transcriptionally suppressing Beclin-1 expression.	(58)
Systemic sclerosis	miR-125b	↓	–	MiR-125b downregulation increased apoptosis, promoted dermal fibroblast proliferation, and increased α-SMA expression.	(87)
	miR-33a-3p	–	DKK-1	MiR-33a-3p down-regulated the expression of DKK-1 in tissues and cells from SSc.	(95)
	miR-5196	↓	fra2	The expression of Fra2 and TIMP-1 was reduced by exogenous transfection of miR-5196.	(91)
	miR-21, miR-29a	↑, ↓	–	The TGF-β-induced fibrotic response in dermal fibroblasts was able to be attenuated after miR-29a expression enhancement or miR-21 activity reduction.	(88)
	miR-29	–	–	CXCL17 post-transcriptionally regulated the expression of type I collagen through miR-29 and MMP1.	(96)
Melanoma	miR-23b	↓	–	MiR-23b up-regulation successfully impaired cell viability and colony formation, inhibited angiogenesis and accelerated apoptosis in SK-MEL-28.	(103)
	miR-214	↑	CADM1	MiR-214 promoted EMT by downregulating CADM1, while miR-214 inhibitor blocked the EMT process.	(107)
	miR-182	↑	RECK	MiR-182 regulated RECK expression and inhibited the proliferation of malignant melanoma cells.	(108)
	miR-495-3p, miR-376c-3p, miR-6730-3p	↑	P2 × 7	Three miRNAs increased the proliferation or migration of melanoma cells.	(112)
	miR-107	–	POU3F2	MiR-107 overexpression reduced migration, proliferation, and invasion of melanoma cells.	(113)
Squamous cell carcinoma	miR-205-5p	↓	TNFAIP8	MiR-205-5p downregulated TNFAIP8-mediated autophagy, increased sensitivity to vemurafenib, and induced apoptosis.	(115)
	miR-21	↑	–	Antagonists of miR-21 rescued GRHL3/PTEN expression levels	(115)
	miR-34a, miR-34b/c	↓	Notch1	Both miR-34a and miR-34b/c inhibited the proliferation, migration and invasion of SCC cells through the Notch1 pathway.	(117)
Basal cell carcinoma	miRNA-451a	↓	TBX1	Overexpression of miRNA-451a significantly inhibited cell growth through G1 cell cycle arrest.	(119)
	miR-18a	↑	Akt, mTOR, Beclin 1, LC3	MiR-18a exerted oncogenic effect.	(120)
Recessive dystrophic epidermolysis bullosa	miR-145p	↑	Unknown	MiR-145-5p demonstrated a profibrotic role in RDEB.	(121)
Systemic lupus erythematosus	miR-31, miR-485-3p	↑	NF-κB, PGC-1α	Overexpression of miR-31 and miR-485-p regulated the production of inflammatory factors, thereby inducing DLE skin inflammation.	(125)

Beyond the diseases discussed herein, miRs are increasingly recognized as key players in a variety of other dermatological conditions, including photoaging, alopecia, pigmentary disorders, acne, and inflammatory dermatoses such as dermatitis and urticaria. Future research should continue to elucidate the specific miR signatures and their functional roles in these prevalent conditions, building upon the mechanistic frameworks and therapeutic concepts explored in this review for other skin diseases.
